# A Test of Energetic Particle Precipitation Models Using Simultaneous Incoherent Scatter Radar and Van Allen Probes Observations

**DOI:** 10.1029/2021JA030179

**Published:** 2022-08-10

**Authors:** Ennio R. Sanchez, Qianli Ma, Wei Xu, Robert A. Marshall, Jacob Bortnik, Pablo Reyes, Roger Varney, Stephen Kaeppler

**Affiliations:** ^1^ Center for Geospace Studies SRI International Menlo Park CA USA; ^2^ Department of Atmospheric and Oceanic Sciences University of California Los Angeles Los Angeles CA USA; ^3^ Center for Space Physics Boston University Boston MA USA; ^4^ Department of Aerospace Engineering Sciences University of Colorado Boulder Boulder CO USA; ^5^ Department of Physics and Astronomy Clemson University Clemson SC USA

## Abstract

Quantification of energetic electron precipitation caused by wave‐particle interactions is fundamentally important to understand the cycle of particle energization and loss of the radiation belts. One important way to determine how well the wave‐particle interaction models predict losses through pitch‐angle scattering into the atmospheric loss cone is the direct comparison between the ionization altitude profiles expected in the atmosphere due to the precipitating fluxes and the ionization profiles actually measured with incoherent scatter radars. This paper reports such a comparison using a forward propagation of loss‐cone electron fluxes, calculated with the electron pitch angle diffusion model applied to Van Allen Probes measurements, coupled with the Boulder Electron Radiation to Ionization model, which propagates the fluxes into the atmosphere. The density profiles measured with the Poker Flat Incoherent Scatter Radar operating in modes especially designed to optimize measurements in the D‐region, show multiple instances of close quantitative agreement with predicted density profiles from precipitation of electrons caused by wave‐particle interactions in the inner magnetosphere, alternated with intervals with large differences between observations and predictions. Several‐minute long intervals of close prediction‐observation approximation in the 65–93 km altitude range indicate that the whistler wave‐electron interactions models are realistic and produce precipitation fluxes of electrons with energies between 10 keV and >100 keV that are consistent with observations. The alternation of close model‐data agreement and poor agreement intervals indicates that the regions causing energetic electron precipitation are highly spatially localized.

## Key Points

- Comparison between observed and modeled density for electron precipitation due to wave‐particle interactions in the magnetosphere
- Comparison verifies the validity of D‐region electron density predicted by pitch‐angle diffusion models of wave‐particle interaction
- Observed electron profiles are obtained with an incoherent scatter radar mode especially designed to optimize measurements in the D‐region

## 1 Introduction

One of the most important and challenging issues in radiation belt physics at the present time is understanding the balance between acceleration of electrons up to relativistic energies and losses out of the system. The precipitation of energetic electrons from the radiation belts into the atmosphere is one of the most important loss mechanisms. It acts as a regulator of radiation belt energy fluxes and, when penetrating into the atmosphere, causes compositional changes to the lower thermosphere, mesosphere and upper stratosphere (e.g., Millan et al., 2013; Turunen et al., 2009).

Current theories of particle precipitation assume that energetic particles in the magnetosphere precipitate into the atmosphere after undergoing pitch‐angle scattering into the loss cone due to interactions with plasma waves near the geomagnetic equator (e.g., Millan et al., 2013; Millan & Thorne, 2007; Thorne, 2010). Several plasma waves are known to be able to scatter electrons into the atmospheric loss cone through pitch angle diffusion. Three wave modes are considered to be particularly dominant in driving this scattering, namely extremely‐low‐ to very‐low frequency (ELF/VLF) whistler‐mode chorus, ELF/VLF plasmaspheric hiss and electromagnetic ion‐cyclotron (EMIC) waves (e.g., Millan & Thorne, 2007). Whistler‐mode chorus waves are discrete whistler emissions observed outside the plasmasphere in the frequency range between the lower hybrid and the electron cyclotron frequencies, *f*_*LHR*_–*f*_ce_ (∼100 Hz–10 kHz), where *f*_ce_ and *f*_*LHR*_ are equatorial electron gyrofrequency and the lower hybrid resonance frequency, respectively. Waves generated at the equator pitch‐angle scatter 0.1–1 keV electrons that generate diffuse aurora (Thorne et al., 2010) and ≳10 keV electrons that generate pulsating aurora (Nishimura et al., 2010). Global surveys of chorus waves near the equator show enhanced fluxes of precipitating electrons with characteristic energies of 10–30 keV in the nightside to dawn sectors for 4 < *L* < 6.5 over a wide range of geomagnetic conditions (Ma et al., 2020). Waves propagating to higher latitude pitch‐angle scatter electrons with ∼100 keV to 1 MeV energies (Horne & Thorne, 2003; Lorentzen et al., 2001). Recently, Miyoshi et al. (2020) proposed a model where whistler waves propagating to high latitudes are responsible for the sub‐relativistic‐to‐relativistic energy microburst electron precipitation in pulsating aurora. Observations of relativistic microbursts associated with pulsating aurora (Kawamura et al., 2021; Shumko et al., 2021) have provided support to the model.

Plasmaspheric hiss is a broadband ELF (100 Hz‐few kHz) whistler mode emission occurring mostly in the high‐density plasmasphere and drainage plumes (Thorne et al., 1973). EMIC waves can occur in three distinct bands: hydrogen band between the He^+^ and H^+^ gyrofrequencies, helium band between the O^+^ and He^+^ gyrofrequencies, and oxygen band below the O^+^ gyrofrequency. EMIC waves are theorized to efficiently scatter relativistic electrons into the loss‐cone through Doppler‐shifted resonances (Albert, 2003; Summers & Thorne, 2003). The importance of EMIC waves as a scattering source of outer radiation belt relativistic electrons has been demonstrated although the scattering efficiency could strongly depend on the modeling parameters (Ross et al., 2020; Zhang et al., 2016).

Experimental verification of the electron atmospheric loss has been focused on correlative studies using particle and optical measurements from the ground, or particle measurements at low‐altitude orbit, and in‐situ measurements of waves and particles in the inner magnetosphere.

Several case studies have revealed a close correlation between electron precipitation, visualized using measurements from ground‐based optical cameras, and chorus waves, measured near the inner‐magnetosphere equator (Hosokawa et al., 2020; Kasahara et al., 2018; Nishimura et al., 2010; Ozaki et al., 2019). Other studies have established correlation between low‐altitude spacecraft observations of electron precipitation and observations of chorus waves near the equator (Breneman et al., 2017; Lorentzen et al., 2001). Studies coordinating ground‐based riometers or balloon‐mounted X‐ray detectors with spacecraft observations have established correlation between chorus waves and Bremsstrahlung X‐ray emissions in the upper atmosphere (Millan et al., 2013; Rosenberg et al., 1971). Observations of microbursts with low‐orbiting spacecraft, coincident with chorus observations near the equator have demonstrated a direct link between relativistic electron microbursts and chorus waves (Breneman et al., 2017; Mozer et al., 2018).

Incoherent Scatter Radars (ISR) have been used previously to measure height‐resolved electron density profiles in the ionosphere associated with pulsating aurorae. Jones et al. (2009) inverted Poker Flat ISR (PFISR) density profiles to infer the energy distribution of precipitating electrons for comparison with in‐situ measurements of electron precipitation energy distributions from rocket and low‐altitude orbit spacecraft. The comparison showed good agreement indicating that electron enhancements in the 90 km region were due to precipitation from pulsating aurora. European Incoherent Scatter (EISCAT) Tromso ISR measurements of density in the lower ionosphere, coordinated with Van Allen Probes measurements of waves and particles were carried out by Miyoshi et al. (2015) to determine whether there is a correlation between the chorus‐induced loss‐cone flux of electrons near the equatorial inner magnetosphere and the electron density observed in the ionospheric D‐ and E‐regions, between ∼68 and ∼190 km. Qualitative similarity between the electron energy spectra in the energy range between ∼10 of keV and ∼100 keV, inferred from inversions of EISCAT radar's electron density measurements averaged over a 22‐min interval, and the averaged spectra observed with a Van Allen Probe suggests that the magnetospheric electron population fills the loss‐cone under the strong‐diffusion limit. Similarity between the same ISR‐inferred energy spectra and the spectra predicted by the by a test‐particle simulation scaled to match Van Allen Probe's particle measurements at the launch point, suggests that whistler chorus is responsible for the observed lower‐energy portion of the energy spectra of precipitating electrons. Recent analysis of EISCAT and Arase data (Miyoshi et al., 2021) compares the energy spectra of precipitating electron flux caused by chorus waves in pulsating aurora, estimated with a test particle simulation of wave‐particle interaction, with the energy spectra estimated from a Monte Carlo inversion of the EISCAT‐observed electron density profiles. The comparison carried out for a time when EISCAT's field‐of‐view was coinciding with an omega band in the pulsating aurora shows a general agreement between the simulation and the inversion calculation with the latter underestimating the former for the lower energy range. Energy spectra comparisons from EISCAT and Van Allen Probes were done applying two inversion methods (Turunen et al., 2016) to estimate ozone depletion in the mesosphere due to energetic particle precipitation and to quantify the error in the high‐energy tail inferred from the inversions. The comparison uses two methods of inversion and shows agreement between both methods and Van Allen Probes for energies between 50 kV and 120 keV. For higher energies both inversions underestimate the Van Allen flux and the uncertainty in the Monte Carlo inversion becomes large due to the uncertainty in the low‐altitude measurements from the radar.

This communication presents the results of a different approach to ISR‐spacecraft comparison. It shows the first direct quantitative comparison between the ISR‐measured electron density profiles and the electron density profiles predicted by a forward electron transport model that propagates the loss‐cone flux, launched by the whistler‐electron interaction near the magnetospheric equator, from the topside ionosphere down to the D‐region. The wave‐particle interaction that predicts the topside precipitating flux is calculated by applying the University of California Los Angeles (UCLA) wave‐particle Full Diffusion Code to Van Allen Probe particle and wave measurements. The density measurements were made with an incoherent scatter radar mode optimized for the estimation of spectra in the collision‐dominated D‐region ionosphere, capable of measurements with sub‐kilometer spatial resolution and ∼1‐min temporal resolution.

The paper is organized as follows. Section 2 describes the three‐step method of analysis. Section 3 describes the characteristics of the storm event where the conjunction develops. Section 4 describes the results of the comparison between observed and predicted density profiles. Section 5 discusses the implications of the comparisons.

## 2 Method of Analysis

The comparison of PFISR‐observed profiles with predicted profiles in each conjunction interval is achieved with a three‐step procedure. The first step is the calculation of loss‐cone electron flux using the UCLA wave‐particle Full Diffusion Code, propagated to the ionosphere at 500 km. The second step is the calculation of the electron density produced by the precipitating flux as a function of altitude using the Boulder Electron Radiation to Ionization (BERI) model. In the final step the electron density altitude profiles observed with the PFISR are compared with the electron density profiles predicted with the BERI model.

*Electron Precipitation Modeling*. In the first step, we use the Van Allen Probes observations and quasi‐linear theory to model the electron loss‐cone fluxes driven by whistler mode chorus waves near the equatorial magnetosphere. The Helium Oxygen Proton Electron plasma spectrometer (Funsten et al., 2013) and the Magnetic Electron Ion Spectrometer (MagEIS) (Blake et al., 2013) instruments measure the electron fluxes at 15 eV–50 keV energies and ∼4.5°–90° pitch angles, and the fluxes at 33 keV–4 MeV energies and ∼8°–90° pitch angles, respectively. The Electric and Magnetic Field Instrument Suite (EMFISIS) instrument (Kletzing et al., 2013) measures the wave electric field and magnetic field intensities and background magnetic fields. The double‐probe electric field instrument obtains two data components perpendicular to the spin axis of the satellite and one component parallel to the spin axis, ranging in frequency from DC to 400 kHz. The magnetometer data has two frequency regimes: the fluxgate, which has frequency coverage from DC to 32 Hz, and the triaxial search coil, which can cover frequencies up to 12 kHz. Both magnetometers are in the same frame of reference as the electric field instruments. The whistler‐mode waves are measured by the Waveform Receiver at frequencies from ∼10 Hz to 12 kHz. The wave polarization properties are provided through the Singular Value Decomposition method, including the wave normal angle, ellipticity, planarity, and degree of polarization. The total electron density is inferred by identifying the upper hybrid resonance frequency line measured by the High Frequency Receiver (Kurth et al., 2015). We automatically select the whistler mode waves by requiring the wave ellipticity to be higher than 0.7 and degree of polarization to be higher than 0.7. During the event analyzed in this paper, the quasi‐parallel and oblique wave components are selected by requiring the wave normal angle to be smaller and higher than 45°, respectively, to account for the different properties of the two groups of chorus waves.

The bounce‐averaged diffusion coefficients are calculated using the UCLA Full Diffusion Code (Ni et al., 2008, 2011), at each time of the whistler mode wave observation by Van Allen Probes. The wave frequency spectrum is obtained from the selected chorus wave intensities. The Van Allen Probes observation of background magnetic field and total electron density are used in the diffusion coefficients calculations. We assume that the wave normal angle distribution follows a Gaussian function, that is, proportional to $\text{exp}\left(-{\left(\frac{\tan\;\theta \;-\;\tan\;\theta_{m}}{\tan\;\theta_{w}}\right)}^{2}\right)$, where *θ*_*min*_ ≤ *θ* ≤ *θ*_*max*_, at the latitude range from equator to the maximum latitude λ_max_. For quasi‐parallel propagating chorus waves, we assume that *θ*_*m*_ = 0°, *θ*_*w*_ = 30°, *θ*_*min*_ = 0°, *θ*_*max*_ = 45°, and *λ*_max_ = 30°; for oblique chorus waves, we assume *θ*_*m*_ = 65°, *θ*_*w*_ = 30°, *θ*_*min*_ = 45°, *θ*_*max*_ = 75°, and *λ*_max_ = 10°. Ten orders of harmonic resonances and Landau resonance are considered (−10 ≤ *N* ≤ 10, where *N* is the harmonic number). We also consider the electron scattering due to Coulomb collision with atmospheric molecules and charged particles (Abel & Thorne, 1998).

Assuming a quasi‐equilibrium pitch angle distribution of electrons, the ratio between the average electron flux inside the loss cone and the flux just outside the loss cone (*χ*(*E*) = *J*_*prec*_/*J*_*out*_) can be estimated using the bounce‐averaged pitch angle diffusion coefficient at the loss cone (〈*D*_*αα*_〉_*LC*_) and the strong diffusion rate (*D*_*SD*_ = 2 ∙ *α*_*LC*_^2^/*τ*_*B*_) as:

(1) $$\chi \left(E\right)=\frac{2\int_{0}^{1}I_{0}\left[Z_{0}\cdot \tau \right]\cdot \tau \cdot d\tau }{I_{0}\left[Z_{0}\right]},$$

where $Z_{0}=\sqrt{D_{SD}/{\langle D_{\alpha \alpha }\rangle }_{LC}}$, *α*_*LC*_ is the pitch angle at the bounce loss cone, *τ*_*B*_ is the bounce period, *I*_0_ is the modified Bessel function of the first kind, and *τ* is an integration variable. The ratio *χ*(*E*) is defined as the loss cone filling index after Ni et al. (2014). The energy spectrum of precipitating electron flux (*J*_prec_) is obtained using the loss cone filling index and the flux just outside the loss‐cone (*J*_out_) obtained from observation. The characteristic precipitating energy *E*_*c*_ is calculated as:

(2) $$E_{c}=\frac{\int_{E_{min}}^{E_{max}}J_{prec}\cdot E\cdot dE}{\int_{E_{min}}^{E_{max}}J_{prec}\cdot dE}.$$

The total precipitating energy flux at the ionosphere is

(3) $$Q=\pi \int_{E_{min}}^{E_{max}}J_{prec}\cdot E\cdot dE,$$

following Liang et al. (2011) and Clark et al. (2018). The modeled average precipitating flux of electrons (*J*_prec_) is mapped to 500 km altitude. We assume an isotropic electron pitch angle distribution within the pitch angle of loss cone, due to the large pitch angle diffusion coefficients induced by intense whistler mode wave scattering and Coulomb collision. The pitch angle scattering rate due to Coulomb collision inside the loss cone is larger or comparable to the strong diffusion limit at energies below tens of keV at *L* = 6. Ma et al. (2021) has shown the consistency between the calculated *J*_prec_ and results from Fokker Planck simulation, after a quasi‐equilibrium state of electron pitch angle distribution is formed near the loss cone.

*Ionization Modeling*. The second step of the procedure is to determine the ionization profiles in the E− and D‐region caused by fluxes and spectra of loss‐cone electrons propagated from the Van Allen Probes' location along the magnetic field to 500 km altitude. The energy and pitch angle distribution of precipitation fluxes determined from the first step are used to calculate the ionization production using the BERI model (Xu et al., 2020). This model is largely based on a lookup table of atmospheric ionization production by monoenergetic electrons with energies between 3 keV and 33 MeV, and pitch angles between 0° and 90°. This lookup table was developed using physics‐based Monte Carlo simulations (Lehtinen et al., 1999), and allows rapid and accurate specification of ionization production by arbitrary precipitation energy and pitch angle distribution in any atmospheric condition. The mass density profile of background atmosphere is calculated using the NRLMSISE‐00 model (Picone et al., 2002) for the date, latitude and longitude of PFISR measurements.

After obtaining the ionization production using the BERI model, we simulate the electron density change at altitudes below 150 km using the Glukhov, Pasko, and Inan (GPI) chemistry model (Glukhov et al., 1992). The GPI model is a five‐species model that includes electrons, heavy and light positive ions, and heavy and light negative ions (Lehtinen & Inan, 2007). This model has been extensively used in studies related to D‐region electron density changes due to transient luminous events or radiation belt precipitation (e.g., Marshall et al., 2019). The GPI code doesn't explicitly model the ion‐ion and electron‐ion recombination reactions as atmospheric chemistry models usually do. Instead, it treats the ionosphere ions as three groups: positive ions, light negative ions (such as O_2_^−^ and O^−^), and heavy negative ions (such as NO_3_^−^). The GPI model then uses an effective coefficient to describe the recombination processes of these cluster ions (the weighted average coefficient calculated from all recombination processes). Despite this simplification, the GPI model has produced good agreements (Marshall et al., 2019) with the Sodankylä Ion and Neutral Chemistry model (Turunen et al., 1996), which has been widely used in ionosphere chemistry modeling. The background ionosphere used in GPI simulation is calculated using the International Reference Ionosphere (IRI) model (Bilitza, 2001), at the date, latitude, and longitude of PFISR measurements.

*PFISR‐BERI Comparison*. The third step is the direct comparison between the electron density profiles inferred with the transport model and the E− and the D‐region electron density profiles measured with the Incoherent Scatter Radar located at the Poker Flat Research Range (65.13°N, 147.47°W). PFISR is a remotely operated, phased‐array radar with pulse‐to‐pulse steering capability (Heinselman & Nicolls, 2008; Nicolls et al., 2007). During expected Van Allen Probes‐PFISR conjunction events, the radar is operated in modes optimized for the estimation of spectra in the collision‐dominated D‐region ionosphere. The spectrum in this regime can be represented as a Lorentzian distribution with increasing spectral width and amplitude as a function of altitude (Dougherty & Farley, 1963; Mathews, 1978; Nicolls et al., 2010). For typical D‐region parameters the spectral width of this type of distribution is between tens and hundreds of Hz, which is much narrower than kHz bandwidths in the E− and F‐region. The narrow bandwidth, which corresponds to a long decorrelation in the time domain, combined with the proximity of the ionospheric target region (∼50–100 km) makes possible the application of pulse‐to‐pulse radar processing schemes. The observations reported here use a 13 baud, 10 μs baud barker code oversampled at 5 μs (750 m spatial resolution). The experiment uses a 2 ms inter‐pulse period, meaning the pulse‐to‐pulse spectra have a Nyquist limit of 250 Hz, and spectra are processed using zero‐padded periodograms of 128 pulses (3.9 Hz spectral resolution). The ion‐line ISR spectrum can only be observed when the radar's Bragg wavelength is long compared to the electron Debye length, and for PFISR operating frequency of 449.3 MHz and a typical D‐region electron temperature of 200 K this limit is encountered at densities of ∼3 × 10^2^ cm^−3^. In practice PFISR does not have sufficient sensitivity to detect densities down to the Debye length limit, and the detection limit varies between 10^3^ cm^−3^ and 10^4^ cm^−3^ depending on the spectral width and target range, with narrower‐bandwidth and shorter‐range targets being easier to detect. Above ∼90 km altitude, frequency aliasing prevents estimation of the spectrum using pulse‐to‐pulse processing, but the total scattered power can still be measured. The D‐region mode used for this study uses four beam positions: vertical (14.0° azimuth, 90.0° elevation), magnetic‐field‐aligned (205.7°,77.5°), and two outrigger beams pointed northwest (325.3°, 66.1°) and east‐north‐east (75.0°, 65.6°), respectively. The magnetic‐field‐aligned beam measurements are used in the present comparisons because the orientation of this beam is closest to the foot‐point of both Van Allen Probes' orbits at the time of the conjunction and it tracks the electron population precipitating along one single L‐shell.

## 3 RBSP‐PFISR Conjunction of 8 May 2018

**Figure 1:**
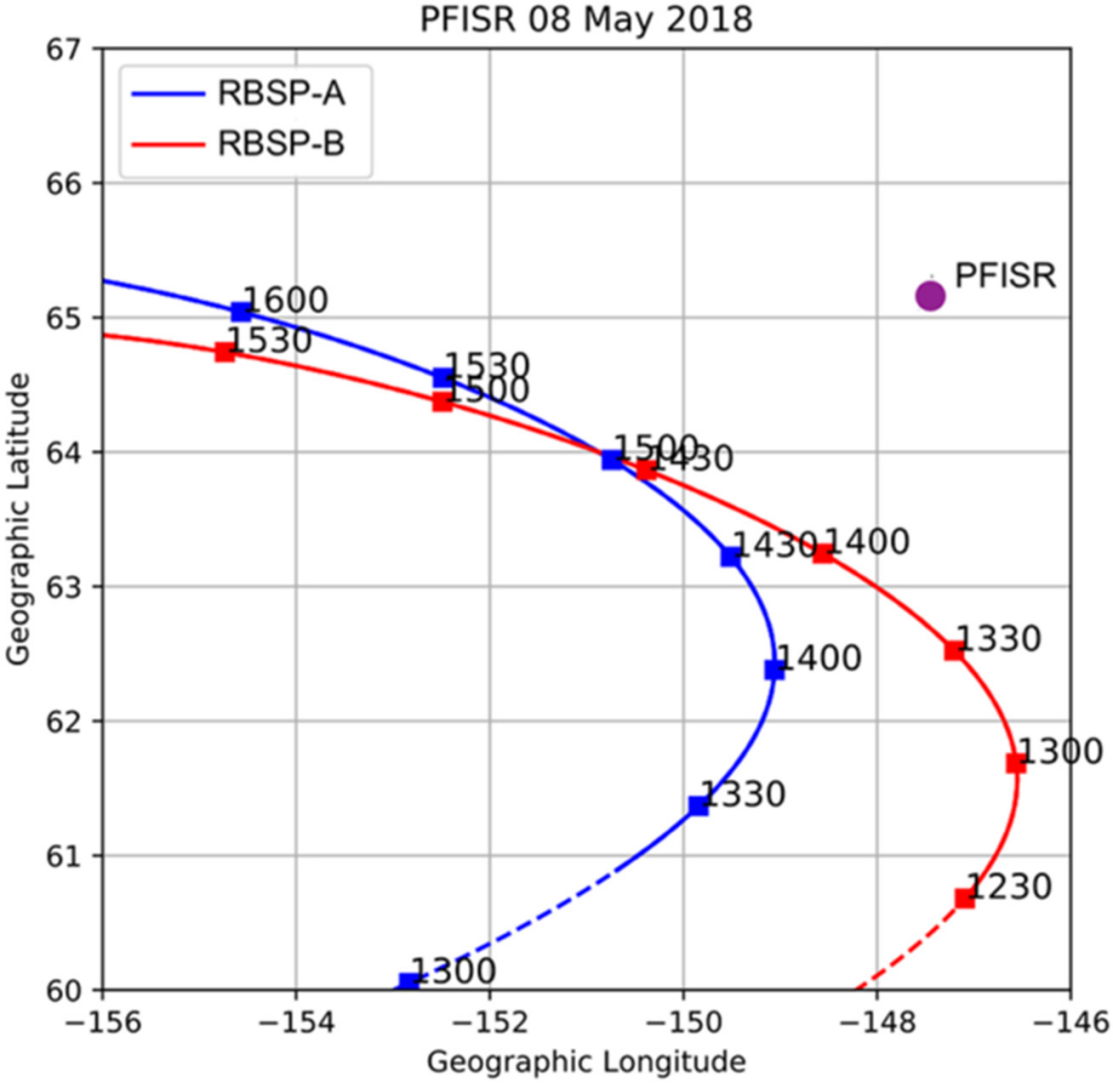
Track of Van Allen Probes orbits' foot‐points (red and blue curves) relative to Poker Flat ISR (PFISR) vertical beam at 100 km altitude in geographic coordinates (purple circle).

The event of interest involves a conjunction between PFISR and both Van Allen Probes in the pre‐dawn sector. During the entire conjunction both Van Allen Probes were near their respective orbit apogee and remained in close proximity to each other, with Probe‐B sampling the same L‐shell and similar MLT approximately 30 min ahead of Probe‐A during 1400–1600 UT (Figure 1). The radar's location projects to a magnetospheric equatorial location of L ∼ 5.3, ∼6.2 R_e_ or ∼6.9, depending on the application of the T89, T02 or T96 Tsyganenko magnetic field models respectively (Tsyganenko, 1989, 1996, 2002) for the moderate storm conditions observed on 8 May 2018 (3 nPa dynamic pressure, 600 km/s solar wind speed, Dst = −30 nT, Kp = 2). Depending on the mapping used, the radar location's projection is either ∼1 R_e_ closer to Earth or ∼0.5 R_e_ farther from Earth than the apogee of the Van Allen Probes (*L* ∼ 6.4). The closest proximity between the probes' foot‐points and PFISR's field‐aligned beam at 100 km altitude is ∼300 km, achieved at 1430 UT for Probe‐B, and ∼350 km at 1445 UT for Probe‐A. However, the situation reverses after ∼1445 UT when Probe‐B's foot‐point starts to have a broader separation from PFISR than Probe‐A.

**Figure 2:**
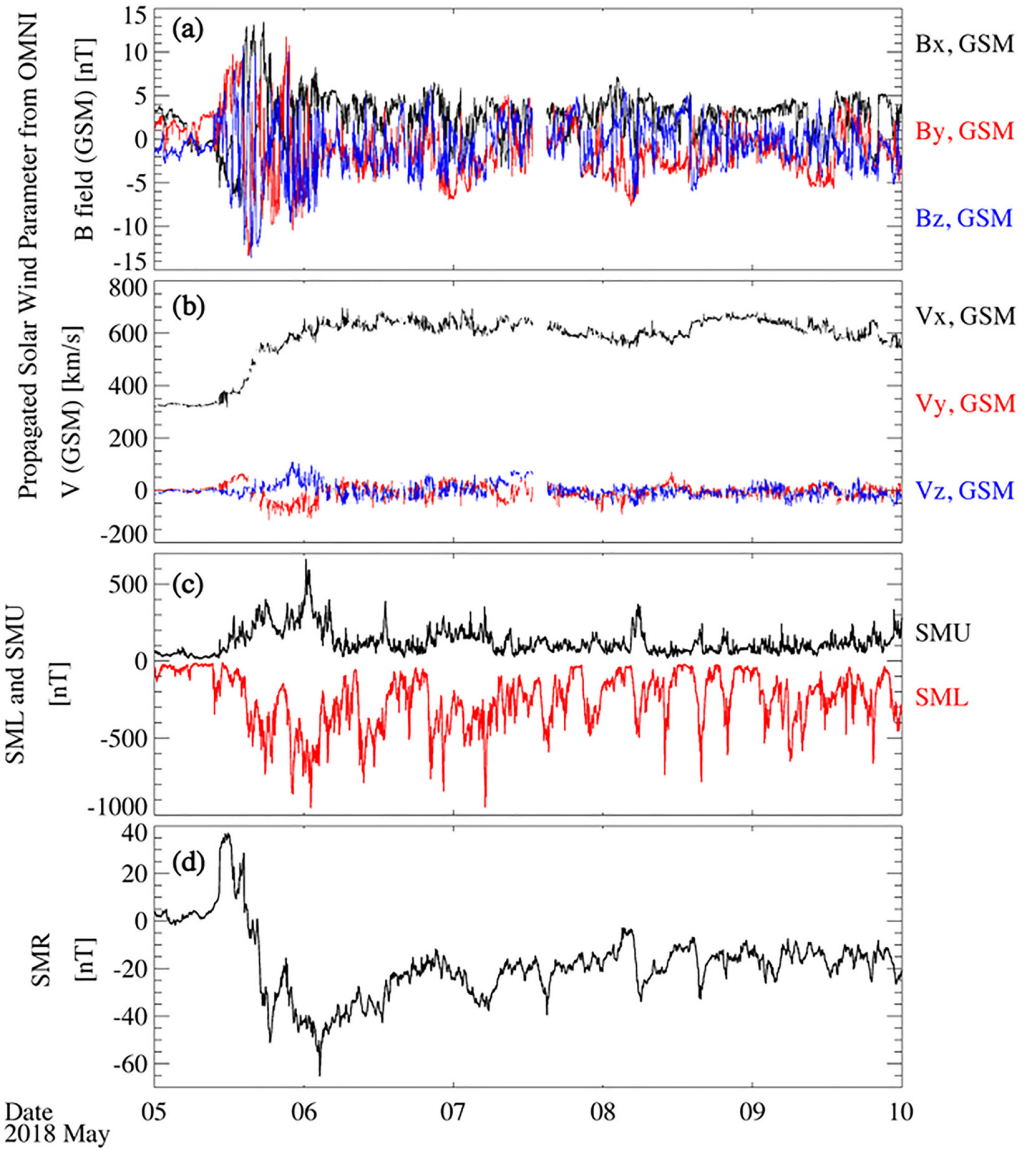
Interplanetary magnetic field (a), solar wind parameters (b), SME U/L indices (c) and SuperMAG Ring current (SMR) index (d) during the high‐speed streamer storm that started on 5 May 2018.

**Figure 3:**
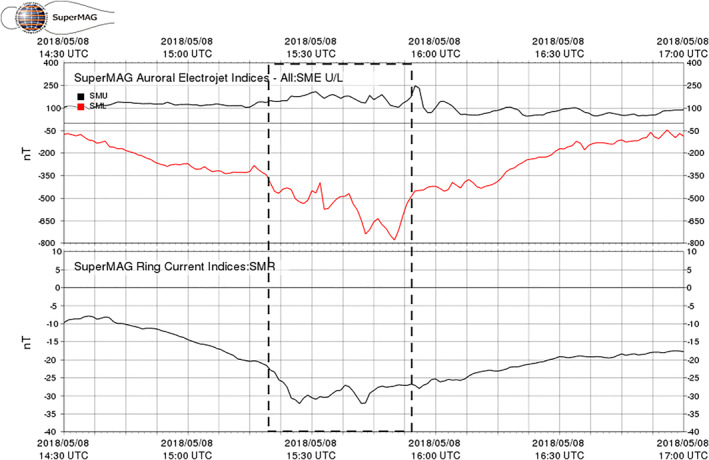
SME U/L indices (top panel) and SuperMAG Ring current (SMR) index for the PFISR‐RBSP conjunction period of 8 May 2018. The dashed box indicates the span of the PFISR‐RBSP conjunction interval analyzed.

The conjunction occurred during a weak storm caused by a high‐speed solar stream and started with a sudden impulse at 1030 UT on May 5 (Figure 2). The storm continued for several days, during which the solar wind speed stayed at ∼600 km/s. The SuperMAG Ring current (SMR) index reached a minimum of −65 nT at the beginning of May 6 and multiple recurring westward electrojet intensifications with SML < −500 nT. The PFISR‐Van Allen Probe conjunction interval occurred during the storm recovery and was embedded in an interval of westward electrojet intensification with a minimum SML (lower envelope of the SuperMAG Auroral Electrojet index) value of −800 nT (Figure 3).

**Figure 4:**
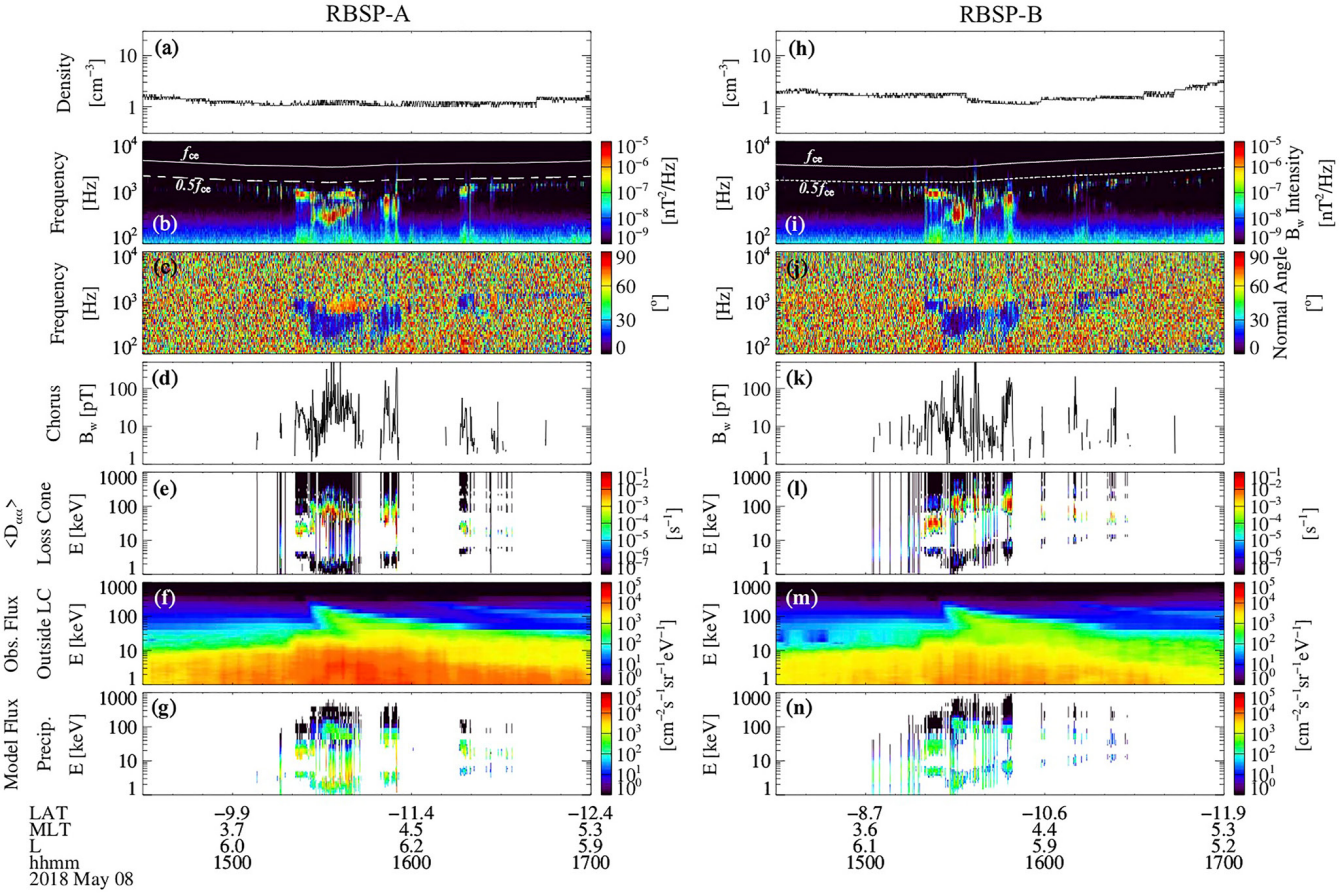
RBSP‐A measurements of (a) total electron density inferred from the upper hybrid line in the HFR spectrogram, (b) magnetic field power spectra with white solid and dashed lines representing equatorial electron gyrofrequency (*f*_ce_) and 0.5*f*_ce_, (c) wave normal angle, (d) chorus wave amplitude, (e) pitch angle diffusion coefficients at the equatorial pitch angle of the bounce loss cone in the 1‐1,000 keV energy range, (f) electron flux in the 1‐1,000 keV energy range using Helium Oxygen Proton Electron below 30 keV and Magnetic Electron Ion Spectrometer above 30 keV, (g) modeled precipitating electron flux from 1 to 1,000 keV using the UCLA full diffusion code. (h–n) RBSP‐B particle and wave measurements and the diffusion modeling results for the same time interval and format.

The Van Allen Probes were located outside the plasmapause as indicated by the low total electron densities (1–3 cm^−3^) observed during 1430–1700 UT (Figures 4a and 4h). Figures 4b and 4i show that the chorus waves observed by both Van Allen Probes spacecraft are very similar to each other. Whistler wave activity in the frequency range from 200 Hz to approximately 2 kHz was observed between 1510 UT and 1640 UT on both spacecraft. The wave power is concentrated in two bands. One at approximately 1 kHz, which is below one half the equatorial electron cyclotron frequency at Van Allen Probes' location, and another that starts at 300 Hz and shifts to higher frequency until it merges with the first band toward 1555 UT. The chorus waves are quasi‐field‐aligned with wave normal angles mostly below 30° (Figures 4c and 4j), except for an oblique wave burst observed during 1525–1540 UT at ∼1 kHz. For the quasi‐parallel propagating chorus waves, we selected the most intense chorus wave power that is expected to effectively precipitate electrons by requiring the wave ellipticity to be higher than 0.7, degree of polarization higher than 0.7, wave normal angle lower than 45°, and wave intensity larger than 10^−8^ nT^2^/Hz. The oblique propagating chorus waves were selected using the same criteria except for requiring the wave normal angle higher than 45°. The largest amplitude of chorus waves reached above 10 pT over all but a few minutes, and peaks above 100 pT intermittently over several minutes, in the interval between 1520 UT and 1555 UT for both spacecraft (Figures 4d and 4k). This interval is the focus of the comparison between predicted and observed electron density profiles.

The pitch angle diffusion coefficients of electrons (Figures 4e and 4l) are calculated using the observed chorus wave frequency spectra. Since the intense chorus waves with small wave normal angles are observed at ∼10° magnetic latitude, we assumed that the latitude range of quasi‐parallel propagating chorus waves is between the equator and 30°. The latitude range of oblique chorus waves is assumed to be between the equator and 10° (e.g., Bortnik et al., 2007). The bounce‐averaged diffusion coefficients reach ∼0.1 s^−1^ when chorus waves are strong, exceeding the strong diffusion limit near the energies of electron cyclotron resonance (e.g., ∼50–100 keV energy during 1530–1540 UT observed by Probe A).

The energy spectrograms of electron fluxes (Figures 4f and 4m) show significant flux enhancement with clear energy dispersion at 20–200 keV energies, indicating an electron injection event occurring simultaneously with chorus wave intensification. The energy spectrum of precipitating electron flux (Figures 4g and 4n) is modeled using the observed electron flux and the diffusion coefficients at the loss cone pitch angle. The most significant electron precipitation is modeled during 1520–1555 UT, and the energy of precipitation varies with the wave frequency. In general, the chorus waves at ∼1 kHz cause the electron precipitation at ∼20–40 keV energies, and the waves at ∼300–600 Hz cause the precipitation at ∼60–200 keV energies.

## 4 PFISR‐UCLA‐BERI Comparisons

**Figure 5:**
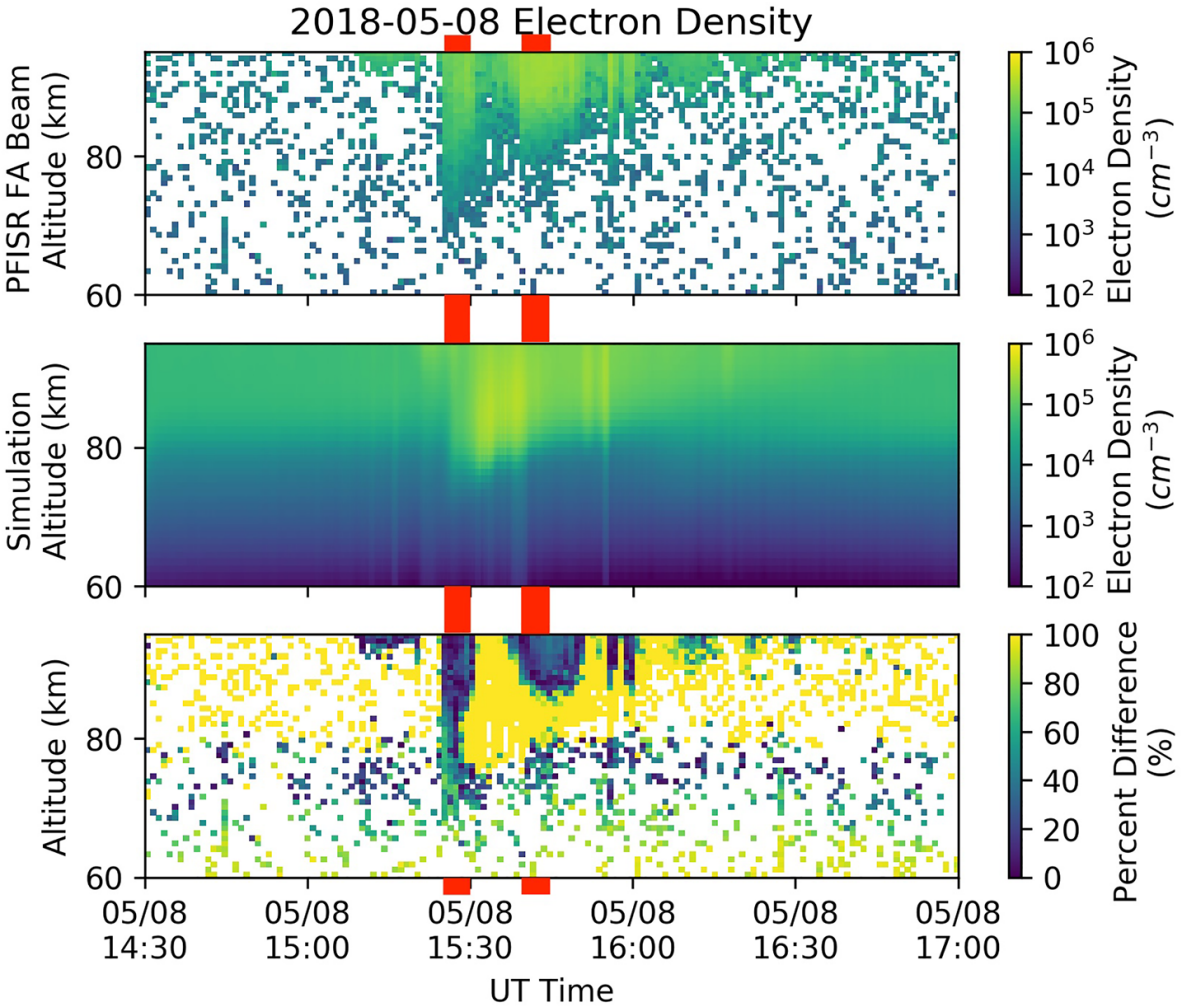
Altitude profiles of electron density measured with the field‐aligned Poker Flat ISR (PFISR) beam (top panel) and UCLA‐BERI‐modeled altitude profiles (middle panel) during the PFISR‐RBSP‐A conjunction period of 8 May 2018. The absolute value of the relative difference between the UCLA‐BERI model and PFISR data is shown in the bottom panel. The left‐side set of red bars indicates the time interval with PFISR‐BERI‐UCLA comparisons in Figure 6. The right‐side set of bars is the time interval for comparisons in Figure 8.

Figure 5 shows a comparison between the estimated electron density (*N*_*e*_) at altitude *h* obtained from fitting the PFISR spectra (top panel) and the *N*_*e*_ predicted from the UCLA‐BERI forward propagation for Van Allen Probe‐A (middle panel), in the entire conjunction interval.

Figure 5 (top) shows the estimated electron density obtained from fitting the PFISR spectra. In the collision dominated D‐region the incoherent scatter spectrum becomes Lorentzian with a spectral width that is inversely proportional to the ion‐neutral collision frequency (Nicolls et al., 2010). The spectra become exponentially narrower with decreasing altitude, which enables easier detection of low electron densities at lower altitudes. The fitting procedure uses a Levenberg‐Marquardt nonlinear least squares algorithm to estimate four parameters for each altitude: electron density (*N*_*e*_), line‐of‐sight Doppler velocity (*V*_*D*_), spectral width (*γ*), and the noise level (*N*). The procedure fits all altitudes simultaneously, and the noise levels are regularized to be close to an a priori measured noise from long ranges. Fitting for the noise level allows the algorithm to compensate for small range‐dependent noise contributions in the radar, range‐aliased F‐region returns, and broad‐band interference. Furthermore, the algorithm uses the full covariance matrix of the calculated spectrum and propagates that covariance into the a‐posteriori error estimates of the fitted parameters. After fitting the incoherent scatter spectra we filter the results based on heuristic acceptance criteria that indicate genuine detections of a Lorentzian IS spectrum. We accept points where *N*_*e*_ > 2*σ*_*Ne*_ and *γ* > 1*σ*_γ_, where *σ*_*Ne*_ and *σ*_γ_ are the a‐posteriori errors of the electron density and spectral width, respectively. The first criterion rejects points that are too small to be statistically significant compared to zero electron density, and the second criterion rejects fits with extremely small spectral widths where the algorithm has likely converged to a local minimum and fit a single point in the digital frequency domain, which is a common problem when fitting Lorentzian spectra to noise‐like data. The white regions in Figure 5 (top) where the fit results have been rejected indicate regions where the electron density is too small for PFISR to reasonably detect. The calculation of electron densities must also involve the assessment of the impact of solar extreme ultraviolet (EUV) on ionization in the mesosphere and lower ionosphere, since the interval of interest occurs shortly before summer solstice. The contribution of solar EUV (1–105 nm) to the ionization rate below 86 km is not comparable to other sources, for example, Lyman‐alpha (see, e.g., Figure 7.12 from Brasseur & Solomon, 2005). Regardless of how much the solar EUV intensity would be enhanced during summer solstice, the lowest altitude that EUV photons can reach is only related to the photon energy, which means the electron density enhancement that PFISR observed below 86 km is mainly caused by precipitation electrons. For our modeling purposes, the background electron density profile was obtained from IRI using the date and location of this event. The effect of solar EUV has already been included in IRI, and the electron density change predicted by BERI is therefore due solely to precipitation electrons.

**Figure 6:**
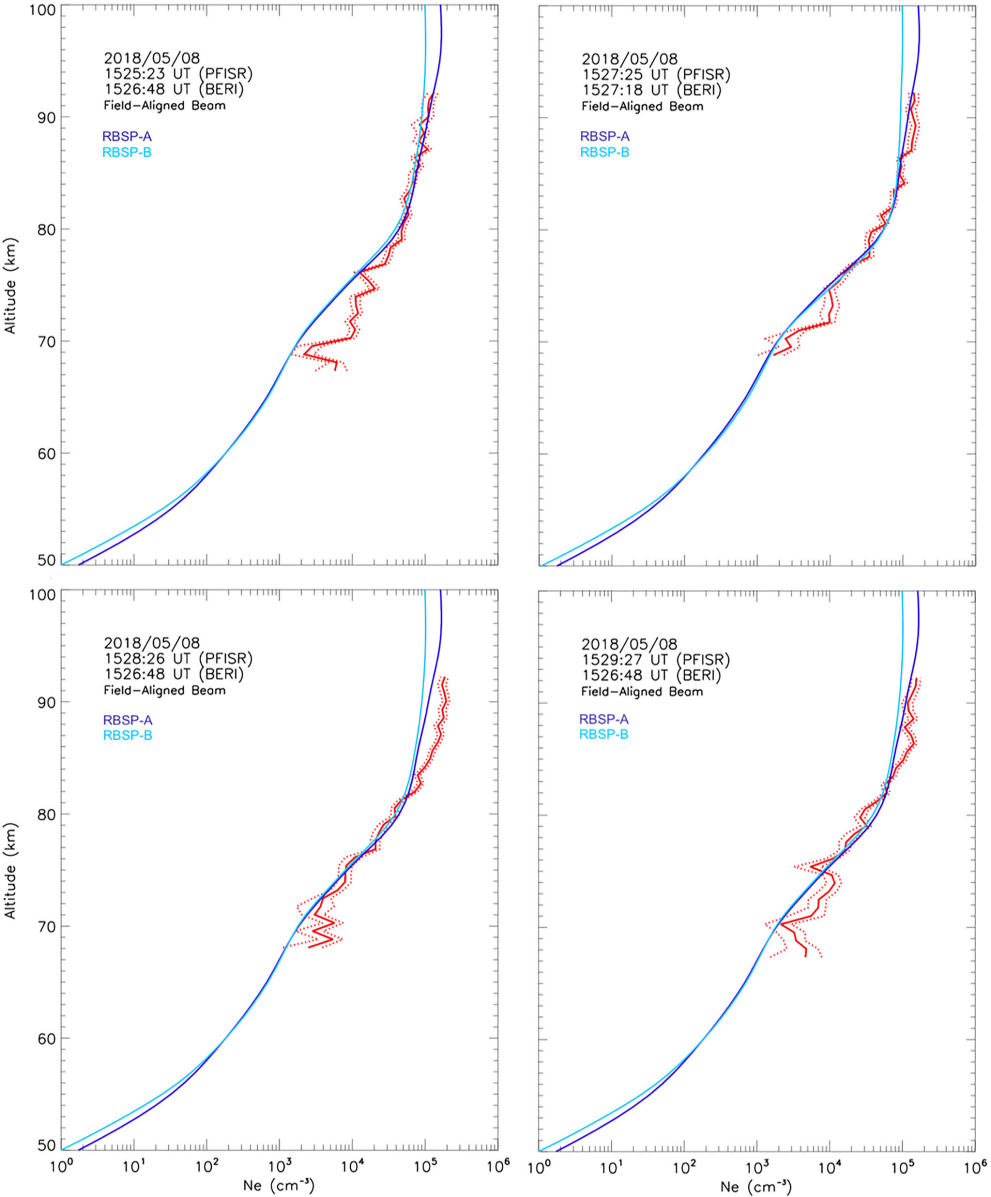
Altitude profiles of electron density measured with the field‐aligned Poker Flat ISR (PFISR) beam (red line) at 1525:23 UT (left top panel), 1527:25 UT (right top panel), 1528:26 UT (bottom left panel), and 1529:27 UT (bottom right panel). UCLA‐BERI‐modeled altitude profile for RBSP‐A (dark blue) and RBSP‐B (light blue) at the start of the energetic particle precipitation interval. Red dotted lines indicate one standard deviation above and below the observed *N*_*e*_ *(h)*.

**Figure 7:**
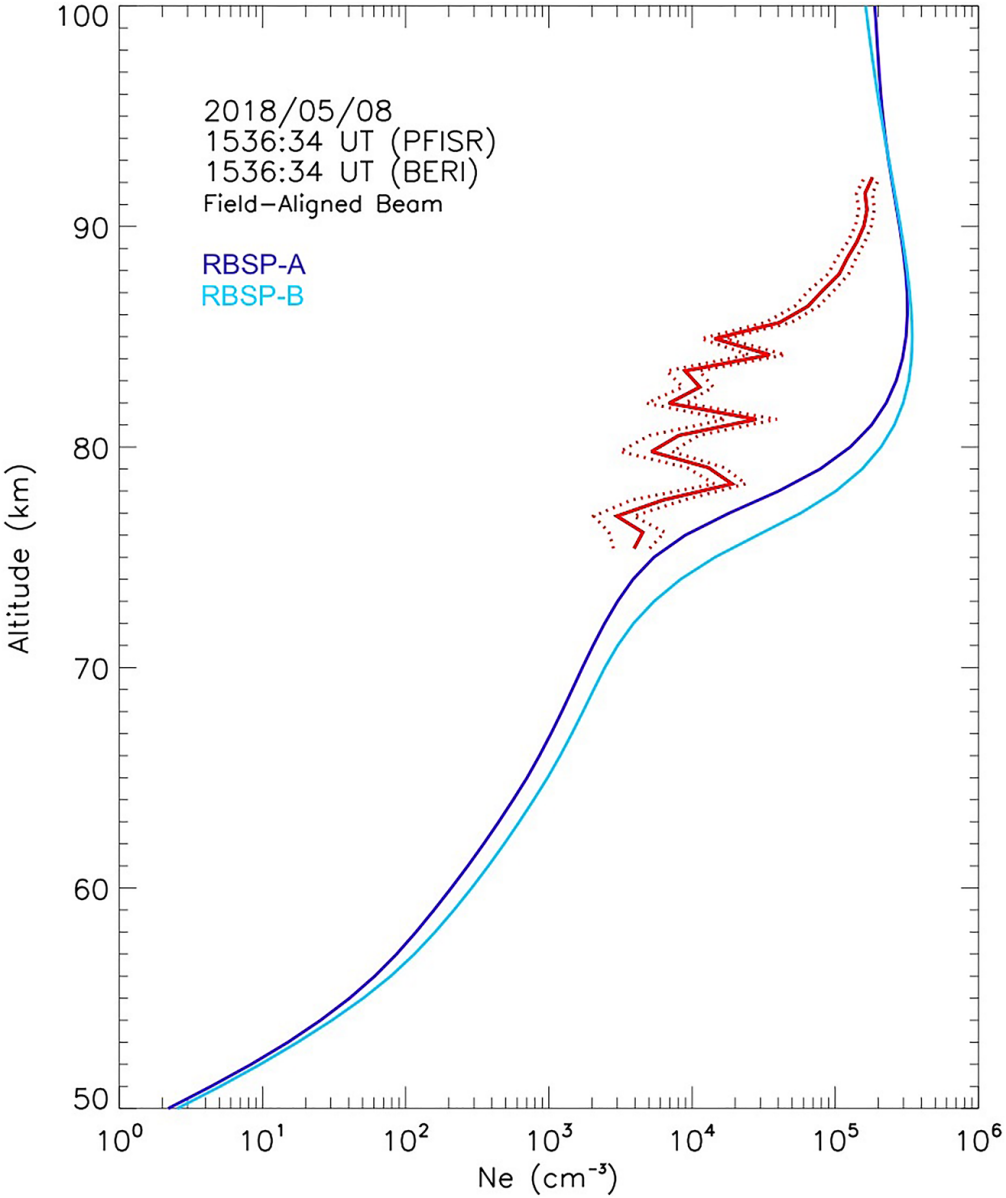
Altitude profiles of electron density measured with the field‐aligned Poker Flat ISR (PFISR) beam (red line) at 1536:34 UT, and UCLA‐BERI‐modeled altitude profile for RBSP‐A (dark blue) and RBSP‐B (light blue). Red dotted lines indicate one standard deviation above and below the observed *N*_*e*_ *(h)*.

A visual comparison between PFISR observations and UCLA‐BERI models' prediction shows the same trends in the properties of the precipitation region. The energetic electron precipitation, characterized by *N*_*e*_ distributions reaching below 80 km altitude, is seen at the onset of energetic precipitation, at 1526 UT, and a nearly monotonic tapering off to lower precipitation energies thereon until 1630 UT, when precipitation occurs above the 93 km upper limit of the PFISR D‐region mode. The bottom panel of Figure 5 shows several intervals of close model‐prediction approximation (dark blue areas) where the relative difference is less than ∼50% and often less than 30%. Two of those intervals are analyzed in detail in this section. Interspersed with these intervals are intervals with large differences between observations and predictions, indicating that the magnetospheric region containing the wave‐particle interactions causing the electron precipitation is either highly spatially localized and intersects PFISR's field‐of‐view every few minutes (there are no optical measurements available to determine whether there is pulsating aurora during this event), or that it is stationary and intensifies intermittently, or both.

A direct model‐data comparison for the first two *N*_*e*_(*h*) 1‐min profiles bracketing the 1526 UT onset of energetic precipitation is shown in Figure 6. Every PFISR 1‐min averaged *N*_*e*_(*h*) profile in the conjunction is compared with consecutive 1‐min averaged *N*_*e*_(*h*) UCLA‐BERI profiles encompassing 10 minutes before and after the PFISR profile until the closest agreement is found for the height range 80 km < *h* < 93 km and the height range 54 km < *h* < 80 km. The ten‐minute criterion allows for a 500 m/s convection in the ionosphere to transport the electron precipitation region the 300 km distance that separates the foot‐point of Van Allen Probe‐A from the PFISR field‐aligned beam. The boundary separating the two altitude regimes is chosen because above 80 km the plasma is dominated by electrons and simple positive ions, but below 80 km the composition transitions to mostly positive cluster ions and negative ions, with free electrons becoming a minor species (Glukhov et al., 1992; Lehtinen & Inan, 2007). Since the 80 km altitude is the peak ionization altitude for 100 keV precipitating electrons (e.g., Xu et al., 2018), it can also be used to separate the high‐energy population from the lower energy plasma sheet electron population, which precipitates at higher altitudes.

For the PFISR profile starting at 1525:23 UT (Figure 6, top left panel), the UCLA‐BERI's results that show best agreement down to 76 km are at 1526:48 UT, after applying model‐data comparison between the 1‐min PFISR integration and every 1‐min model‐predicted profile in the ±10‐min window, indicating that a good agreement occurs at approximately the same time for PFISR and Van Allen Probes. The agreement degrades for the higher energy precipitation, corresponding to altitudes between 70 and 76 km. The measured density is ∼4.5 times as large as the expected density at 71 km for Van Allen Probe‐A. The high‐energy model‐data discrepancy is significantly reduced for the 1527:25 PFISR profile and its best agreement from the models' results of 1527:18 UT (Figure 6, top right panel). The discrepancy is reduced to a layer between 71 and 74 km. The observed density is ∼3 times the predicted density at 72 km.

Subsequent modeled 1‐min profiles maintain good approximation to observations but with progressively more time delay between PFISR and Van Allen Probes, indicating that it takes longer for the region of precipitation observed by the Van Allen Probes to reach the precipitation region sampled by PFISR. The 1528:26 UT PFISR profile (Figure 6 bottom left panel) is best approximated by the modeled precipitation from 1526:48 UT between 68 and 82 km. But in the 82–93 km altitude layer the observed *N*_*e*_ is larger than the model‐predicted *N*_*e*_. It is as much as ∼58% larger at 87 km for Van Allen Probe‐A. The discrepancy is reduced to ∼48% and to the 84–88 km altitude layer in the next 1‐min profile (Figure 6, bottom right panel), although the profile approximation is slightly degraded below ∼74 km.

The model‐data discrepancy becomes more pronounced for all subsequent altitude profiles between 1529:42 UT and 1537:34 UT, with PFISR‐measured densities significantly smaller than predicted for all sampled altitudes, as shown in Figure 5. In that interval PFISR is inside a region of depleted electron density while both Van Allen Probes spacecraft are in a region that generates an electron density peak at ∼83 km that is not reproduced by any of the PFISR *N*_*e*_ profiles measured within the sliding ±10‐min window that would fit any convection delays or drift front geometries. The predicted density shows an intense peak between ∼2 × 10^5^ cm^−3^ and ∼4 × 10^5^ cm^−3^ over an altitude between ∼83 and ∼85 km which is not reproduced in the PFISR measurements during the entire density depletion period. Figure 7 shows the UCLA‐BERI‐predicted density profile at 1536:34 UT, with a peak altitude of 85 km and a density of 3.5 × 10^5^ cm^−3^. The observed density is lower at every measured altitude (becoming over one order of magnitude smaller at 85 km) and the density peak is at least 7 km higher.

**Figure 8:**
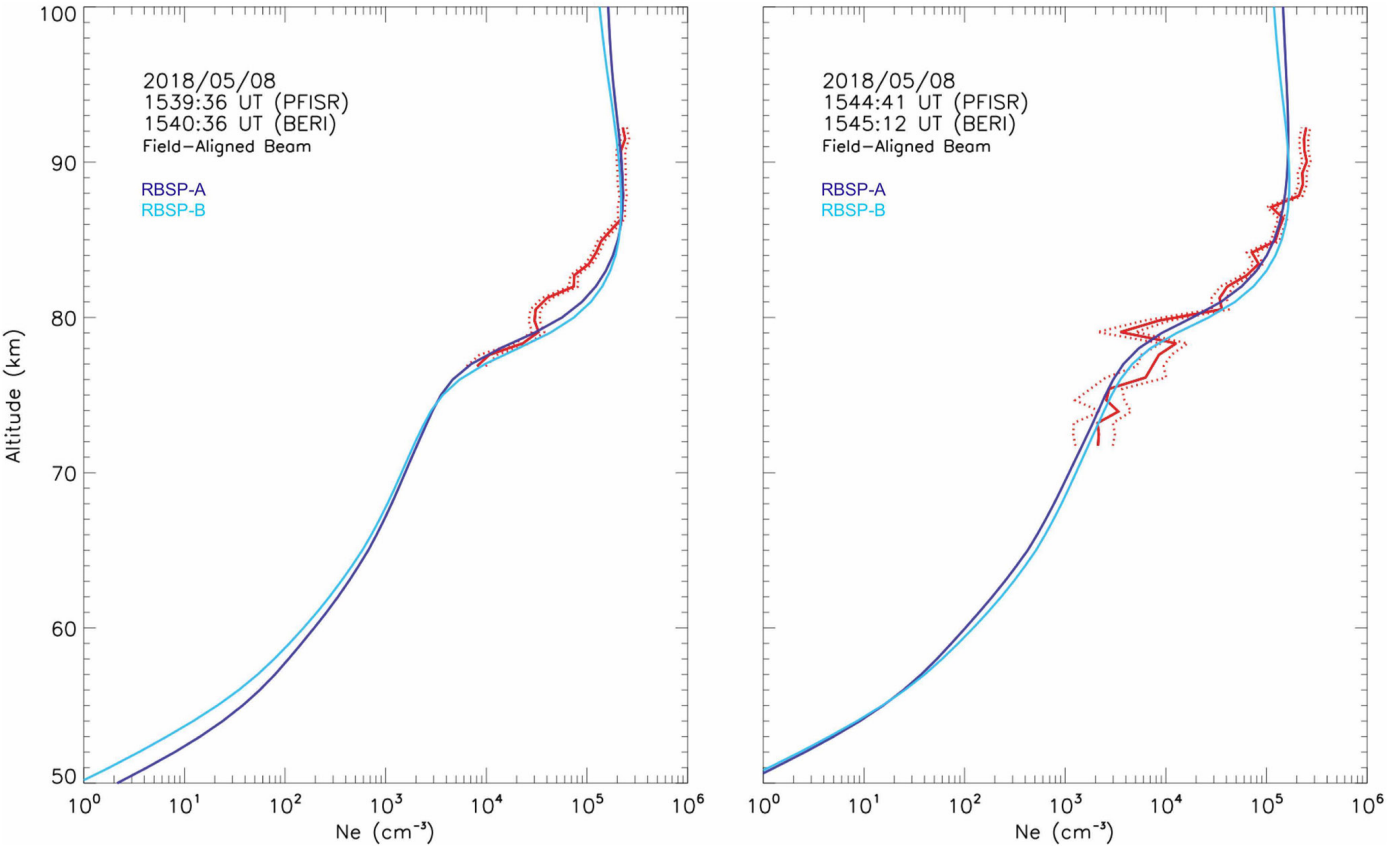
Altitude profiles of electron density measured with the field‐aligned Poker Flat ISR (PFISR) beam (red line) at 1539:36 UT (left panel) and 1544:41 UT, and UCLA‐BERI‐modeled altitude profile for RBSP‐A (dark blue) and RBSP‐B (light blue). Red dotted lines indicate one standard deviation above and below the observed *N*_*e*_ *(h)*.

Model‐observation agreement becomes stronger after 1537:34 UT. At 1539:36 UT, for instance, the PFISR‐observed profile matches UCLA‐BERI‐predicted profile below 80 km and above 85 km (Figure 8, left panel). For the intermediate altitudes the observed density shows a deficit of ∼50–60% near the peak of the profile. However, subsequent profiles show a close model‐observation agreement over a wider range of altitudes, as can be seen in the 1544:41 UT comparison (Figure 8, right panel), where the good fit extends between 71 and 93 km, with the exception of a 77–80 km altitude layer where PFISR‐measured density is 1.5 times smaller than the modeled density, and an 88–93 km layer, where PFISR‐measured density is 35% larger than the modeled density. In both cases the closest model‐data agreement occurs when the region of precipitation reaches PFISR slightly before it reaches the Van Allen Probes.

## 5 Discussion and Conclusion

This communication reports the first direct comparison between the ionization altitude profiles expected in the atmosphere due to energetic electron precipitation and the ionization profiles measured with incoherent scatter radars. The comparison is performed using a forward propagation of loss‐cone electron fluxes, calculated with the UCLA wave‐particle model applied to Van Allen Probes measurements, into the topside ionosphere and transported into the atmosphere with the BERI model.

One of the two principal results of the prediction‐observation comparison is that density profiles measured with PFISR show multiple instances of close quantitative agreement with predicted density profiles from precipitation of electrons caused by whistler wave‐electron interactions in the inner magnetosphere.

There are two intervals between ∼1524 UT (the onset time of high‐energy electron precipitation commences) and ∼1550 UT (the time when precipitation recedes above the upper boundary of sensitivity of the PFISR D‐region mode) where close approximations of UCLA‐BERI‐predicted electron density profiles and PFISR‐measured density profiles are apparent in the entire altitude regime between ∼65 and ∼93 km, indicating that the rates of diffusion into the loss‐cone predicted by the UCLA Full Diffusion Code produce the necessary amount of flux for all energies between several tens to several hundreds of keV to match the ionization profiles observed at those altitudes by PFISR. Good model‐observation agreement is apparent despite the assumptions embedded in the models and the spatial separation between the field‐of‐view of the field‐aligned PFISR beam and the ionospheric projections of the Van Allen Probes' locations at the time of the observations. There are several intervals in the observation period that show a close prediction‐observation agreement over nearly the entire range of altitudes sampled (see bottom panels of Figures 6, 7, 8) indicating that the diffusion of local electrons into the loss‐cone caused by interaction with upper and lower band whistler chorus causes sufficient flux to explain the D‐region electron density enhancements observed. However, there are other instances where better approximations are possible above 80 km or below 80 km but not simultaneously. This discrepancy indicates that the low‐frequency whistler bands, responsible for scattering electrons with higher energy, and the high‐frequency bands, responsible for lower energy precipitation, have a different duration and or different characteristic spatial scales.

Although UCLA‐BERI‐calculated density profiles undergo changes at various altitudes, the evolution of profiles calculated for Probe‐A is replicated for Probe‐B with better than 20% approximation in most altitudes and times and no more than 50% difference in the cases of largest difference at altitude above ∼80 km.

The other main result of the present analysis is that the intervals of close model‐data proximity are interspersed with intervals where there is a distinct discrepancy between measured density profiles and density predicted by the forward model. The alternation of good with poor approximations indicates that the magnetospheric region containing the wave‐particle interactions causing the electron precipitation is either highly spatially localized and intersects PFISR's field‐of‐view every few minutes, or that it is stationary and intensifies intermittently, or both. The frequent close proximity of the in‐situ wave and particle properties observed by both Van Allen Probes spacecraft, which translates into similarity of *N*_*e*_ profiles calculated from the precipitating electrons, shows that the spacecraft are often embedded in the same magnetospheric region and that the region has a spatial coherence of at least 1574 km, which is the in‐situ separation between the two Van Allen Probes at 1550 UT. This distance is within the ∼5,000 km coherent scale size estimated for pulsating aurora (Nishimura et al., 2011) and approximately one order of magnitude smaller than the separation between either probe and the projection of PFISR into the magnetic equator using T96 magnetic field tracing. The corresponding inter‐spacecraft separation in the ionosphere (130 km) is between two and three times smaller than the separation between PFISR's field‐aligned beam and the foot‐point of Probe‐A (290 km) and Probe‐B (390 km), respectively, with PFISR's location always north of either spacecraft's location. The distance between the radar's beam and either spacecraft foot‐point is 3–4 times the size of the coherent scale size projected to the ionosphere. The relatively large separation between either spacecraft and the projection of PFISR's flux tube is thus the likely reason for the lack of reasonable model‐data agreement in the interval between 1529:42 UT and 1537:34 UT. The observed density is consistently lower than the predicted density in that interval. A plausible reason for the discrepancy during some intervals and quantitative approximation for others is that the region of wave‐particle interactions causing electron precipitation is a highly spatially localized region that only intermittently overlaps the Van Allen Probes and the PFISR flux tube simultaneously.

The instances of close model‐data agreement indicate that, despite the spatial separation, PFISR may be measuring the effects of the whistler‐electron interaction at the edge of the precipitation region that is being sampled by the Van Allen Probes. When the region's boundary moves earthward because of a temporal or spatial shift of the precipitation region, PFISR's flux tube migrates to the lower density region just outside. If PFISR is instead measuring the effects of an entirely separate region, then the close model‐data approximation indicates that the wave properties at two adjacent regions are very similar.

## Data Availability

Poker Flat ISR data are available through the AMISR data access repository https://amisr.com/amisr/links/data‐access/. Van Allen Probes wave and particle data are available through the Van Allen Probes Science Gateway https://rbspgway.jhuapl.edu/. Results from the UCLA diffusion code are available at https://doi.org/10.6084/m9.figshare.16942696. Results from the Boulder Electron Radiation to Ionization Monte Carlo simulations are available at https://doi.org/10.5281/zenodo.5651509.
